# Influence of Sex on Gestational Complications, Fetal-to-Neonatal Transition, and Postnatal Adaptation

**DOI:** 10.3389/fped.2018.00063

**Published:** 2018-04-23

**Authors:** Sheila Lorente-Pozo, Anna Parra-Llorca, Begoña Torres, Isabel Torres-Cuevas, Antonio Nuñez-Ramiro, María Cernada, Ana García-Robles, Maximo Vento

**Affiliations:** ^1^Neonatal Research Group, Health Research Institute La Fe, Valencia, Spain; ^2^Neonatal Research Group, Division of Neonatology, Hospital Universitari i Politècnic La Fe, Valencia, Spain; ^3^Division of Neonatology, Hospital Universitari i Politècnic La Fe, Valencia, Spain

**Keywords:** sex, fetal-to-neonatal transition, prematurity, resuscitation, vascular reactivity, antioxidant defenses

## Abstract

Fetal sex is associated with striking differences during *in utero* development, fetal-to-neonatal transition, and postnatal morbidity and mortality. Male sex fetuses are apparently protected while *in utero* resulting in a higher secondary sex rate for males than for females. However, during fetal-to-neonatal transition and thereafter in the newborn period, female exhibits a greater degree of maturation that translates into a better capacity to stabilize, less incidence of prematurity and prematurity-associated morbidities, and better long-term outcomes. The present review addresses the influence of sex during gestation and postnatal adaptation that includes the establishment of an adult-type circulation, the initiation of breathing, endurance when confronted with perinatal hypoxia ischemia, and a gender-related different response to drugs. The intrinsic mechanisms explaining these differences in the perinatal period remain elusive and further experimental and clinical research are therefore stringently needed if an individual oriented therapy is to be developed.

## Introduction

**Figure 1:**
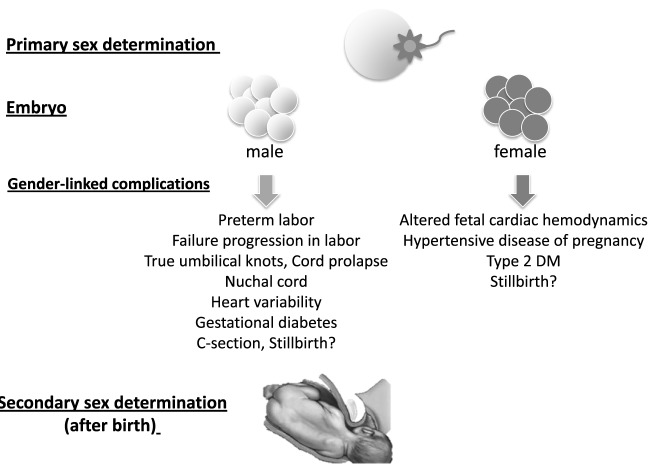
Primary sex rate is determined at birth. However, along gestation different circumstances modify survival of fetuses from different gender and therefore secondary sex rate is that present at birth.

Vital statistics from different countries point out that the number of male born infants overcomes that of females. In 1997, the sex ratio male vs. female after studying a population of 549,048 births was established in 1.06% (1). The human sex ratio is thought to be the result of two processes (Figure 1). First, the sex of the zygotes is significantly influenced by the hormonal activity of the progenitors during the periconceptional period. Second, maternal stress induces the production of adrenal androgens leading to selective spontaneous abortion of male sex embryos. Both these circumstances are relevant conditioning factors contributing to the secondary sex ratio at birth. However, studies trying to unveil the factors that determine the sex ratio at birth and to identify associations between sex ratio and other perinatal circumstances such as still birth, and/or birth or parental-related factors have yield inconclusive results (2). In developing countries where the incidence of prenatal losses and stillbirths is significantly greater than in developed countries, male/female sex ratio at birth has been established around 102 (3).

The sex ratio at conception or primary sex ratio (PSR) in humans is unknown. No scientific evidence, however, has supported the assumption that PSR is more male-biased than the birth sex ratio (secondary sex ratio). In previous studies, estimates of the PSR have been claimed to be around 0.56 (proportion of males) or even greater. Statements supporting that PSR are unbiased, it is slightly male or female biased, or that it cannot be estimated due to an absence of sound data or methodological issues have been evenly called upon (4). Orzack et al. analyzing 3–6 day embryos, estimated the sex ratio (male proportion) at conception in 0.5. Interestingly, the sex ratio of abnormal embryos is male biased while normal embryos are female biased. Bias is associated with the Y and X chromosomes and of chromosomes 15 and 17. Along gestation, the sex ratio varies. After an initial increase in male mortality, an increased female mortality follows thereafter finally overcoming male mortality at the end of gestation. This would explain why the secondary sex ratio for males is greater that for females. In the neonatal period, however, male mortality and morbidity significantly overcomes that of females (4).

In 2013, the leading causes of neonatal death worldwide were attributed to prematurity-associated and birth-related complications such as birth asphyxia or trauma, infections, and congenital malformations. Although the order of relevance is highly dependent on the level of income, cultural and social development of each country in every country and for all diagnostic groups the proportion of affected males was significantly higher than that for females (3, 5). Recently, United States vital statistics from 2013 informed that the overall infant mortality rate for male newborn infants as compared to female was 6.51 vs. 5.39 per 1,000 births, 21% higher coincident with other relevant reports (6, 7).

In 1971, the term “male disadvantage” was coined by Naeye et al., referring to the higher incidence of perinatal mortality for male as compared to female newborn infants. Interestingly, in the analysis of 2,735 consecutive newborn autopsies, these authors found that the rate of males to females was 1.28:1. Male tendency toward increased morbidity and mortality was related to conditions associated with birth. Hence, removal of the male fetus from maternal utero predisposed to conditions that increased morbidity and mortality thus unveiling male-inherent biological disadvantages when confronted with the extrauterine milieu (8). The exact mechanisms involved in male biologic disadvantages remain unclear; however, a body of evidence reveals that obstetric risk factors such as hypoxia, the influence of sex hormones, alterations in cell death pathways and sensitivity to inflammation and excitotoxins, as well as sex differences in autonomic and endocrine stress responses seem to play a relevant role in this biologic difference between males and females (9).

The aim of the present review is to inform how the sex of the fetus influences morbidity and mortality during pregnancy and the neonatal period.

## Sex Rate and Pregnancy Outcomes

The association of fetal sex with gestational complications such as preeclampsia, preterm delivery, glucose tolerance, spontaneous abortion, stillbirth, or complications during delivery such as true cord knots, alteration of fetal heart variability, need for C-section among others have been largely assessed in clinical studies and recently reviewed (10). Hence, Sheiner et al. (11) compared 50,000 pregnancies with male fetus vs. a similar number with female fetus. Interestingly, pregnancies with female fetuses had a higher incidence of preeclampsia and glucose intolerance while pregnancies with male fetuses had a significantly higher incidence of prematurity and complications such as macrosomia, failure of progress during the first stages of labor, cord prolapse, among other factors contributing to interfere with eutocic vaginal delivery, thus concluding that male sex is an independent risk factor for delivery-associated adverse pregnancy outcomes (Table 1). In addition, in chromosomally normal spontaneous abortions the female sex ratio was 1.32 representing a 30% increased risk ratio for male fetuses (12). The national medical birth registry of Sweden, which included gestations of ≤28 weeks if the infant was alive at birth showed no difference in the incidence of stillbirth between male (3.8/1,000) and female (3.9/1,000) fetuses. However, the number of male newborn infants dying in the neonatal period or before 1 year of age was a greater than 50%. The rate of mortality for males at 1 year was 3.44/1,000 and 2.18/1,000 for females (13).

**Table 1:** Odds ratio (OR) for males regarding gestational conditions that alter fetal progression in labor after Sheiner et al. (11).

Condition	OR	Significance
Macrosomia	2.0 (1.8–2.1)	<0.001
Failure to progress labor	1.2 (1.1–1.3)	<0.001
Cord prolapse	1.3 (1.1–1.6)	<0.014
Nuchal cord	1.2 (1.1–1.2)	<0.001
True umbilical cord knot	1.5 (1.3–1.7)	<0.001
C-section	1.2 (1.2–1.3)	<0.001
5 min Apgar score	1.5 (1.3–1.8)	<0.001

### Hypertensive Disorders of Pregnancy

Fetal sex has been linked to hypertensive disorders pregnancy, especially preeclampsia. Preeclampsia is associated with an increased risk for both maternal and fetal morbidities and mortality. In two large studies, Shiozaki et al. (14) and Zheng et al. (15) found that carrying female fetuses in singleton, monochorionic diamniotic, and dichorionic diamniotic pregnancies was significantly associated with pregnancy-induced hypertension. They concluded that female fetal sex was a risk factor for both pregnancy-induced hypertension and preeclampsia (14, 15).

### Prematurity

Preterm birth remains the major cause of infant mortality worldwide with a complex and multifactorial etiology with potentially interacting causes (16). Preterm labor is considered idiopathic inasmuch as 40% of cases while the remaining 60% is associated with preterm premature rupture of the membranes or maternal and/or fetal infection. Maternal risk factors include, obesity (body mass index > 25), previous preterm deliveries or abortions, cesarean section, the use of reproductive techniques, twin pregnancy, physical extenuation, and previous preterm birth and ethnicity. These proportions may vary with gestational age but also with the economic and social development of the countries (17, 18). National figures from 1999 to 2000 in the national medical birth registry from Sweden showed an increased number of males born between 24 and 37 gestational weeks. However, the number of females overcame that of males between 38 and 40 gestational weeks (13). In a register study that included almost two million births performed in New England (US), an excess of 7.2% males among white singleton preterm births over 20–37 weeks of gestation was found; however, when black singleton preterm incidence was assessed only 2.8% excess was found (19). Similarly, Wilms et al. found that gestational age at birth of pregnancies of male and female fetuses did not significantly differ; however, in Caucasians, they found a significantly increased risk for preterm delivery before 37 weeks in women pregnant with male fetus odds ratio (OR) 1.9 (95% CI 1.2–3.0) (20).

## Fetal-to-Neonatal Transition

### Delayed Lung Antioxidant Defense System Maturation

Early in gestation sex, differences in lung development become already apparent. During the canalicular and initial phase of the saccular stage, the lungs of female fetuses are more structurally mature when compared to their male counterparts. However, differences fade along the late saccular stage and disappear by 32 weeks gestation. Of note, most preterm neonates cared for in the neonatal intensive care units (NICU) pertain to the range of 24th to 32nd weeks’ gestation, precisely when the difference in lung development is more apparent. This should be taken into consideration by the attending neonatologists. Interestingly, the growth of lung parenchyma significantly correlates with airway growth in males while other factors, such as genetics, are more relevant for airway growth in females. These differences become apparent, and despite having larger lung volumes, young males have decreased forced expiratory flows and other respiratory functions after birth than females. These functional characteristics seem to be associated with increased smooth muscle and thicker airway walls in males while females have larger central airways and thus airway resistance (21).

In the fetal-to-neonatal transition, the initiation of breathing abruptly increases oxygen availability eliciting the generation of reactive oxygen species (ROS). ROS act as signaling molecules that stimulate the expression of specific metabolic pathways. However, preterm infants frequently need oxygen to achieve postnatal stabilization. In the presence of immature antioxidant system, oxygen supplementation causes a burst of oxygen free radicals, pro-oxidant imbalance, oxidative stress, and tissue damage (22). The “free radical disease of the newborn period” coined by Saugstad included the most relevant conditions in the newborn period such as bronchopulmonary dysplasia (BPD), retinopathy of prematurity (ROP), persistent ductus arteriosus (PDA), intra-periventricular hemorrhage (IPVH), and necrotizing enterocolitis (NEC) (23). Classical studies from Frank and Sosenko revealed that the maturation of the antioxidant defense system in different experimental models in mammals occurred late in gestation paralleling that of alveolar surfactant (24). In a prospective observational study performed in extremely preterm infants (<28 weeks’ gestation) administration of antenatal steroids to the mother produced a significant increase in the activity of the antioxidant enzymes, glutathione redox cycle enzymes, and reduced to oxidized glutathione ratio reflecting an enhanced response to perinatal oxidative stress. However, antioxidant and clinical response to steroids was significantly lower in males than in females as reflected by increased urinary ortho-tyrosine/phenylalanine and 8-hydroxy-2′-deoxyguanosine/2′-deoxiguanosine ratios in males. Clinical outcomes such as BPD, ROP, IPVH, NEC, or PDA were significantly better in female preterm infants during the neonatal period. The degree of maturation of females amounted over 1 week that of male infants as deduced from the response to antenatal steroids and activity from antioxidant enzymes (25).

### Pulmonary Circulatory Changes at Birth

**Figure 2:**
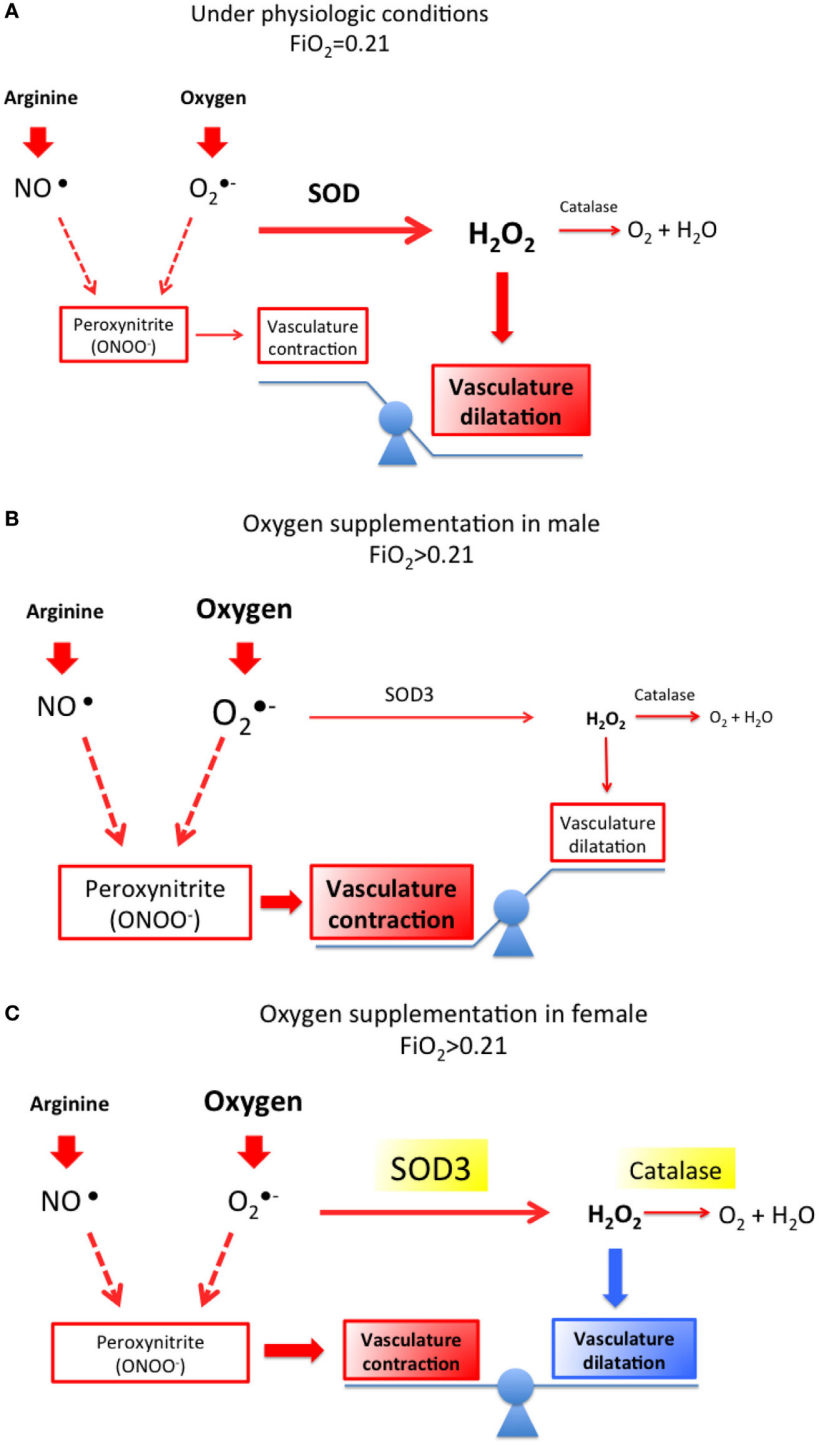
**(A)** Under normal circumstances, the vasodilating action of oxygen and nitric oxide (NO) and other vasodilating agents overcome the vasoconstrictive action triggered by oxygen (superoxide) and nitrogen free radicals (peroxynitrite) through the action of superoxide dismutases (SOD3). **(B)** However, supplementation with high oxygen concentration may lead to an increased generation of free radicals that may overcome the neutralizing effect of SOD3 (extracellular SOD) and lead to vasoconstriction. **(C)** Enhanced expression of SOD3 in females is highly protective toward the development of pulmonary vasoconstriction at birth especially in preterm infants.

During fetal life gas, exchange takes place in the placenta. Both the elevated pulmonary vascular resistance (PVR) and the low placental vascular resistance present in the fetus diverts a large blood volume away from the lungs toward the placenta. The high PVR during the fetal period is the result of a combination of mechanical factors such as the presence of fluid filling the airways and alveoli, vasoconstrictor (endothelin 1 and thromboxane) and vasodilator mediators’ interactions [nitric oxide (NO) and prostacyclin], and relative hypoxemia. Immediately after birth, a series of anatomical and physiological changes lead to the clearance of the airways. Coinciding with birth, there is an involution of the medial smooth muscle of the pulmonary arteries and the thinning of the small pulmonary arteries and with the intense initial respiratory movements, there is the generation of high negative intra-thoracic pressure. Altogether, these factors contribute to the intrusion of lung fluid into the interstitial tissue thus clearing the airways. In addition, elevation of alveolar oxygen tension also favors pulmonary vasodilation, elevation of arterial partial pressure of oxygen, and establishment of an adult-type cardiopulmonary circulation (26–28). However, an adequate oxygenation of the pulmonary vessels is required to adequately adjust the vascular tone. Pulmonary vasodilation is highly dependent on the activation of the NO and prostacyclin (PGI2) pathways (Figure 2A) (29). However, brief hyperoxia secondary to high FiO_2_’s provided to preterm infants or asphyctic term babies may contribute to the generation of mitochondrial ROS and especially hydrogen peroxide (H_2_O_2_) a signaling molecule that induces critical changes especially in NO specific pathway (30). H_2_O_2_ activates phosphodiesterase 5 (PDE5) in fetal pulmonary artery smooth muscle cells. PDE5 degrades cyclic GMP (cGMP) and inhibits NO-mediated cGMP-dependent vasorelaxation (Figure 2A) (31). Experiments performed in different animal models have shown that long-lasting supplementation with oxygen leads to structural and functional damage in lungs and other organs (32). Remarkably, sex-related differences in the rodent survival rates have been assessed following long-term exposure to oxygen; thus, female rodents were more tolerant than their male counterparts. Remarkably, castration of young male rats caused an increased tolerance to chronic hyperoxia for as yet unknown reasons (33). Enomoto et al. (34) showed (Figures 2B,C) that also a brief oxygen exposure simulating that of the delivery room had significant effects upon vascular tone during the immediate postnatal transition in a newborn rat model. Moreover, vascular response was highly dependent on the sex of the rats. Hence, following 1-h exposure to 100% oxygen, pulmonary arteries and lung tissue were evaluated. Superoxide dismutase (SOD) expression in female pup’s lungs was greater than in males. In addition, the vasoconstrictor effect of thromboxane in male pups was increased by oxygen, whereas the opposite effect was documented in female pups. The increased vasoconstrictor effect of oxygen in male pups was abolished with the incubation with SOD or peroxynitrite scavengers. In addition, both increased lung SOD activity and H_2_O_2_ were seen in female, but not in male, rats. Hyperoxia caused the generation of lung tissue oxidation byproducts and increased the activity of Rho-kinase (ROCK) in males but not in female pups (34). This is in agreement with previous findings in a clinical study performed in extremely preterm infants by Vento et al. who showed that antioxidant enzyme activities (SOD, catalase, and glutathione peroxidase) were significantly higher in female preterm than in male independently of the mothers receiving or not antenatal steroids (25). In females, the increased activity of SOD dismutates excess of superoxide anion generated by hyperoxia to H_2_O_2_, thus reducing the formation of peroxynitrite and its vasoconstriction effect and favoring vasodilation while in male anion superoxide will bind with NO enhancing the formation of peroxynitrite and secondary vasoconstriction (Figures 2B,C) (34).

### Breathing After Birth

Numerous experimental studies have shown delayed maturation of lung structure and function in males that undoubtedly hinder postnatal adaptation. The administration of antenatal steroids in a sheep model caused a significantly greater improvement in lung function and oxygenation in females than in males (35). Male rats tend to respond with pulmonary vasoconstriction as compared to female rats that respond with vasodilation (34). In addition, while estrogens contribute to lung barrier formation, alveolar development and surfactant production testosterone has an inhibitory effect. Thus, female lambs responded to inflammation caused by the instillation of lipopolysaccharides with significantly greater increase in lung gas volumes than did males (36, 37).

Human female neonates also show enhanced lung maturation for similar gestational age than do males favoring postnatal adaptation and reducing the incidence of immaturity-associated lung conditions. During fetal development, female fetuses have shown to produce surfactant earlier, have greater swallow activity, develop larger airways that are more resistant to insult, and achieve a more mature lung (21). Consequently, striking differences in lung function and incidence of acute and chronic lung conditions between preterm boys and girls have been found. Hence, compared with boys, maximal expiratory flow at functional residual capacity was considerably higher whereas respiratory resistance was notably lower in girls (38). In a prospective observational study performed in preterm infants <32 weeks gestation breathing with face mask and air during postnatal stabilization, Vento et al. found that preterm females achieved significantly earlier targeted preductal saturations than males (39). In the NICU, males have a higher incidence of respiratory disorders and behave from a respiratory perspective as if they were 1–2 weeks younger than their female correlates with similar gestational age. Hence, the incidence of respiratory distress syndrome and chronic lung disease is significantly higher in males (21). Elsmén et al. in a retrospective study retrieved for 5 years’ data from 236 patients (130 male and 106 female) <29 weeks’ gestation and found significant sex-related differences. Hence, more males than females (60.8 vs. 46.2%) required mechanical ventilation, more males required more doses of surfactant, and also males (40). A study performed in the Canadian Neonatal Network between 2000 and 2005 also showed an increased prevalence of BPD in male infants born at 24–26 weeks gestation thus revealing that male preterm infants are at higher risk of respiratory complications especially in the lowest gestational ages (41).

### Birth Asphyxia and Hypoxic Ischemic Encephalopathy (HIE)

Perinatal asphyxia is one of the most important pathogenic factors leading to neonatal neurologic morbidity and mortality and a leading cause of long-term neurocognitive and sensorial dysfunction among survivors. The prevalence of HIE among term newborn infants is 1–4/1,000 in industrialized countries but can reach significantly higher incidence in non-industrialized countries. Around 20–50% of infants with HIE will die in the early neonatal period and 25–60% of the survivors will suffer from long-lasting neurologic disabilities that include among others cerebral palsy (CP), seizures, behavioral and learning defects (42). Mohamed and Aly retrieved data from the Nationwide Inpatient Sample Database, which comprises over 1,000 hospitals in the United States. They included babies >36 weeks gestation and >2,500 g at birth and excluded babies with severe congenital malformations or chromosomal disorders. Birth asphyxia in males and females was compared. After examining >9 million registries, the OR for severe asphyxia in male newborn was 1.16 (CI: 1.12–1.20; *p* < 0.001) (43). These results coincide with a recent meta-analysis that showed that male infants have greater long-term IQ impairment than females with a similar degree of HIE (44). One of the most relevant complications of birth asphyxia, which is the most frequent cause of motor deficiency in childhood, is CP. A network of CP surveillance in Europe retrieving registries from multiple centers in eight countries conferred CP a prevalence of in 2–3 per 1,000 live births (45). Among the predisposing factors for CP pregnancy-induced hypertension was the most common antenatal complication while prematurity and birth asphyxia were the most common postnatal complications. Remarkably, sex distribution coinciding with the European Surveillance was male to female ratio of 1.2 (46).

Although clinical predisposition to gestational and labor complications have been widely reported (8–21), the intrinsic mechanisms leading to enhance brain damage associated with birth asphyxia, prematurity, or perinatal infections remain elusive. Experimental research of neonatal hypoxia ischemia (HI) performed in rat models has revealed a greater susceptiveness to behavioral and neurocognitive deficits in males as compared to females with a similar degree of brain damage. Hence, pro-apoptotic signaling pathways and caspase-independent cell death tendency are strikingly different between males and females (47). Notably, the pathophysiology of perinatal brain damage of different etiologies (asphyxia, prematurity, and infection) is tightly linked to hypoxia-reoxygenation that entail alteration of mitochondrial function that leads to the generation of highly ROS such as anion superoxide and hydroxyl radical that cause oxidative stress, alteration of the redox code, and activation of transcription factors NF-κ B, AP-1, p53, HIF-1α, PPAR-γ, β-catenin/Wnt, and Nrf2 (30). Recent experimental studies have evidenced that following HI mitochondrial respiratory activity was significantly more damaged in males than in females. Moreover, males endogenous glutathione reserves, the most relevant cytoplasmic non-enzymatic antioxidant, were substantially lower, exhibited a decreased glutathione peroxidase activity following HI injury. Under these circumstances, male rats were significantly more susceptible to HI as shown by increased content of oxidation byproducts such as protein carbonyl in different areas of the brain (48). Of note, female rats highly express the mitochondrial biogenesis-associated transcription factor Nrf2/GABPα following HI while males do not. In the presence of free radicals, Nrf2 translocates into the nucleus and binds to the DNA antioxidant responsive elements promoting the expression of multiple antioxidant defense related genes (30). As a consequence, there is an increase in the electron transport chain proteins that could partially explain the increased resistance of females to respiratory impairment and secondary neuronal damage (49).

### Sex and Response to Drugs

The influence of sex in the response to drugs has only been recently studied [for a review see Ref. (50)]. Traditionally sex was not taken into consideration as a confounder in clinical trials. However, its inclusion as a decisive variable in the statistical analysis of trials’ results has contributed to explain the different response to drugs individuals depending on their sex (51). In the experimental setting, animal models have shown sex differences in the response to drugs. Hence, experimental studies on the effectiveness of vasoactive drugs to overcome the loss of cerebral autoregulation in a traumatic brain injury piglet model have shown striking differences between males and females. Hence, after provoking a moderate fluid percussion brain injury, the protective autoregulation response to drugs such as phenylephrine, norepinephrine, and dopamine was age and sex dependent. These results underscore the need for specific targeted pharmacotherapy that takes into consideration additional factors such as postnatal age and sex (52).

Few clinical studies have reported sex differences in the neonatal period. Ohlsson et al. performed a subset analysis in the course of a multicenter randomized controlled trial in extremely low-birth-weight infants found that the prophylactic use of indomethacin prophylaxis slightly favored male regarding development of severe IVH (grades III and IV) and on long-term outcomes (53). In a recent study, human umbilical artery smooth muscle cells were employed to analyze sex differences in basal and drug-induced autophagy a process, which is involved in cardiovascular diseases. Cells were isolated from healthy male and female newborn umbilical cords. Constitutive autophagy was similar in both sexes; however, after starvation promoted autophagy increased in both sexes but was significantly higher in females. Moreover, response to rapamycin was exclusively present in females. Contrarily, no sex difference was found when verapamil was tested. These studies clearly show that sex differences already begin *in utero*. Moreover, they are parameter specific and drug specific (54).

## Conclusion

Although the PSR is equal in male and female embryos, there is a tendency toward increased survival of males *in utero*. However, functional and structural development of the lungs and regulation of cardiorespiratory circulation are substantially more mature in females and therefore they are capable to better face the difficulties inherent to fetal-to-neonatal transition and postnatal adaptation especially in babies born prematurely. As a consequence, intact survival in the neonatal period is significantly higher in female than in male infants. Sex-related response to drugs is a fertile field for the development of individually targeted therapy in the next future.

## Author Contributions

SL-P researched and reviewed the literature and contributed to the draft of the manuscript. AP-L researched and reviewed the literature especially that related with neonatal pharmacological aspects and that related with sex, and contributed to the draft of the manuscript. BT researched and reviewed the literature, retrieved statistics of the NICU, and contributed to the draft of the manuscript. IT-C contributed to the drawing of the figures and to the draft of the manuscript. AN-R contributed to the draft of the manuscript. MC reviewed the final version of the manuscript. AG-R researched and reviewed the literature and contributed to the draft of the manuscript. MV critically reviewed the literature and approved the final version of the manuscript.

## Conflict of Interest Statement

The authors declare no conflicts of interest or disclosures in relation to the present manuscript.
